# Experiences of Inpatient Healthcare Services Among Children With Medical Complexity and Their Families: A Scoping Review

**DOI:** 10.1111/hex.14178

**Published:** 2024-09-04

**Authors:** Tammie Dewan, Lyndsay Mackay, Lauren Asaad, Francine Buchanan, K. Alix Hayden, Lara Montgomery

**Affiliations:** ^1^ Department of Pediatrics University of Calgary Calgary Alberta Canada; ^2^ College of Nursing Trinity Western University Langley British Columbia Canada; ^3^ Patient, Family and Community Engagement, The Hospital for Sick Children Toronto Ontario Canada; ^4^ Library and Cultural Resources University of Calgary Calgary Alberta Canada

**Keywords:** children with medical complexity, communication, experience of care, inpatient, relationships

## Abstract

**Background:**

Children with medical complexity (CMC) have high healthcare utilization and face unique challenges during hospital admissions. The evidence describing their experiences of inpatient care is distributed across disciplines. The aim of this scoping review was to map the evidence related to the inpatient experience of care for CMC and their families, particularly related to key aspects and methodological approaches, and identify gaps that warrant further study.

**Methods:**

This scoping review was conducted in accordance with JBI methodology and included all studies that reported experiences of acute hospital care for CMC/families. All study designs were included. Databases searched included EMBASE, CINAHL Plus with Full Text, Web of Science, MEDLINE(R) and APA PsycInfo from 2000 to 2022. Details about the participants, concepts, study methods and key findings were abstracted using a data abstraction tool. A thematic analysis was conducted.

**Results:**

Forty‐nine papers were included: 27 qualitative studies, 10 quantitative studies, six mixed methods studies, two descriptive studies and four reviews. Some quantitative studies used validated instruments to measure experience of care, but many used non‐validated surveys. There were a few interventional studies with a small sample size. Results of thematic analysis described the importance of negotiating care roles, shared decision‐making, common goal setting, relationship‐building, communication, sharing expertise and the hospital setting itself.

**Conclusion:**

CMC and families value relational elements of care and partnering through sharing expertise, decision‐making and collaborative goal‐setting when admitted to hospital.

**Patient or Public Contribution:**

This review was conducted in alignment with the principles of patient and family engagement. The review was conceptualized, co‐designed and conducted with the full engagement of the project's parent–partner. This team member was involved in all stages from constructing the review question, to developing the protocol, screening articles and drafting this manuscript.

## Practitioner Points

Qualitative research describes key elements of inpatient experience of care for CMC and their families as relationships, communication, respect for parent/provider expertise and the hospital setting. There is less evidence on measuring experience of care using quantitative methodology, particularly using validating instruments. This would be helpful to support the evaluation of inpatient interventions to improve the experiences of CMC.

## 1 Introduction

Children with medical complexity (CMC) have among the highest inpatient utilization in paediatrics, accounting for up to half of the children admitted to tertiary care paediatric hospitals [1, 2]. CMC share characteristics of chronic diseases, high healthcare utilization, high care needs in the home and community and functional limitations [3]. They are one of the fastest growing populations in paediatrics and collectively account for one‐third of child health spending [4, 5]. Considerable efforts have focused on determining the best models of care to support these children [6, 7]. Much of this work has focused on outpatient care coordination programmes. However, as one of the highest utilizers of inpatient resources, evaluating and designing inpatient care models is an equally important priority.

CMC are at increased risk of adverse events and experiences during hospitalizations. Numerous factors complicate the care of CMC in hospital including polypharmacy, rare diseases, need for multispecialty and multidisciplinary care, complex technology and inherent fragility [8, 9]. Their hospitalizations are more likely to be prolonged and they have higher rates of readmissions than other paediatric groups [10, 11]. They are more likely to need intensive care and have higher rates of death [12, 13, 14]. Adverse events and medication errors are more common among CMC [11, 15, 16]. CMC and their families are less satisfied with inpatient care than either children with acute illnesses or single non‐complex chronic diseases [17, 18].

The Institute for Healthcare Improvement framework for value‐based care emphasizes patient experience as one of the core aims [19]. Experience of care includes all interactions, shaped by an organization's culture, that influence an individual's perception of the care they receive [20, 21]. This includes functional elements such as wait times as well as relational elements such as communication with healthcare providers (HCPs) [22]. This outcome shows consistent positive associations with patient safety, adherence to treatment regimens and objectively measured health outcomes [22]. The patient and family experience of care includes both their perceptions and ratings of the care delivered; thus, it can be influenced by unique personal circumstances and past experiences [23, 24].

Perhaps not surprisingly, CMC and their families often rate their experience of care lower than other patient groups [24]. This could be related to their greater frequency of adverse outcomes. However, qualitative research points towards other potential contributors. Parents who provide high levels of expert care in the home may struggle with a lack of control in the hospital setting [25]. HCPs do not have a template for integrating parental expertise into inpatient care [26]. The need to negotiate roles between expert parents and HCPs can introduce additional stress and tension [27]. Finally, the fragility of these children and the frequency of their hospitalizations cannot be underestimated as sources of stress to families [28, 29].

Improving the care of CMC in the hospitalized setting is an emerging focus in the field of paediatric hospital medicine. A Canadian national James Lind Alliance (JLA) priority setting partnership in 2020 emphasized the need to identify best practices and models of inpatient care for CMC [30]. Developing these models will require close attention to all pillars of care—patient safety, clinical effectiveness and experience of care—to produce the greatest impact. Scientific evidence describing experience of care for CMC could reveal the most important pressure points but is currently distributed across disciplines. Further, studies to date are limited by small sample sizes of specific populations in discrete clinical settings, which likely do not reflect the breadth of experience of CMC. If synthesized, this evidence could be an essential foundation to developing new models of care.

### 1.1 Aim and Objectives

The aim of this scoping review was to synthesize the evidence related to the inpatient experience of care for CMC and their families to guide the future development and evaluation of inpatient health services for this population. Specifically, the review objectives were to (a) describe and map existing evidence including key aspects of experience of care and methodological approaches and (b) identify gaps in knowledge that warrant further study.

## 2 Methods

A scoping review was chosen as the most appropriate method to map the existing literature around a concept, as opposed to answer a specific research question, particularly when the evidence is broad and diverse [31, 32]. This review followed the JBI approach to scoping reviews and is reported in accordance with the Preferred Reported Items for Systematic Reviews and Meta‐Analyses Scoping Review (PRISMA‐ScR) statement [33, 34]. The protocol was developed a priori and published in Open Science Framework [35].

### 2.1 Search Strategy

The search was constructed with the guidance of an academic health sciences librarian (KAH). Databases searched included EMBASE (OVID), CINAHL Plus with Full Text (EBSCO), Web of Science Core Collection, MEDLINE(R) and Epub Ahead of Print, In‐Process, In‐Data‐Review & Other Non‐Indexed Citations and Daily (OVID) and APA PsycInfo (OVID). Keywords and subject headings were developed for each of the two main concepts, ‘CMC’ and ‘inpatient care setting’, that formed the basis of the review. The search was limited to publications from 2000 onwards corresponding broadly to the release of the landmark Institute of Medicine report highlighting the importance of patient experience of care as an outcome and quality indicator [21]. Searches were conducted on 26 April 2022, and the complete search strategies for all the databases can be found in Supporting Information S1. Search results were uploaded into Covidence, a screening and data extraction software, where duplicates were removed and all screening activities took place.

### 2.2 Inclusion and Exclusion Criteria

*Participants*: This review included CMC (age 1–17 years) as well as their parents, family members and caregivers. Alternate descriptors were included in the search (e.g., children with complex, chronic conditions). To qualify for the review, at least 50% of the participants in a study must meet these criteria. After the review began, studies were found that included both a CMC population and a non‐CMC control group. These studies were felt to be eligible for inclusion because CMC were the population of interest, even if the comparator group was larger. Qualitative findings from non‐CMC/control populations were not incorporated into the results of this review.

*Concept*: This review focused on the concept of ‘experience of care’, defined as the sum of all interactions, shaped by an organization's culture, that influence patient perceptions across the continuum of care [36]. This pertains specifically to experiences as a ‘user’ or consumer of healthcare services [20]. Some published definitions of experience of care also include ‘human’ and ‘illness’ experiences, as described by Oben and Corliss [37]. The definition chosen for this review focused on user experiences, but human/illness experience of care will be analysed and reported in a future manuscript.

*Context*: Studies that pertained to the inpatient setting, particularly in acute care hospitals (ward and intensive care), were included.

*Types of sources*: Quantitative, qualitative, experimental and observational study designs and reviews were all included. Grey literature, letters to the editor and opinion papers were not included.

*Exclusion criteria* included care settings of palliative care, neonatal intensive care unit (NICU) and inpatient mental health. These were felt to be unique settings that would warrant a more targeted review. Infants less than 1 year were excluded as a recent review addressed this population [38]. Studies specifically addressing the experiences of hospital discharge were also excluded, as this topic was felt to warrant a separate review.

### 2.3 Screening and Selection

The inclusion and exclusion criteria were first pilot tested independently by all reviewers. The team then met to discuss disagreements and clarify the selection criteria. Following the calibration exercise, four reviewers participated in the screening where two independent reviewers screened each record. Disagreements were resolved by discussion. A third reviewer was available to help resolve disagreements but was not required.

### 2.4 Data Abstraction and Synthesis

The data extraction tool was created by reviewers and pilot tested on the first three records by two independent reviewers. No modifications were necessary. The data extracted included specific details about the participants, concepts, context, study methods and key findings. A final calibration step was performed in which a second reviewer reviewed the abstraction of the first five records. There was satisfactory agreement, so one reviewer proceeded to extract the remainder of the data. The data were synthesized in text and tables.

### 2.5 Thematic Analysis

Findings were analysed using inductive thematic analysis [39]. Qualitative results from these studies included current experiences as well as participants' reflections on past experiences (positive and negative) and ‘ideal’ experience of care. Incorporating the spectrum of these results into our analysis contributed to a comprehensive understanding of experience of care, the definition of which includes the subjective perceptions and perspectives of individuals. One research team member performed the primary coding of the qualitative findings. Preliminary themes were presented to the full research team (with representation from multiple health disciplines and a parent–partner) at regular intervals to discuss and refine themes.

## 3 Results

**Figure 1:**
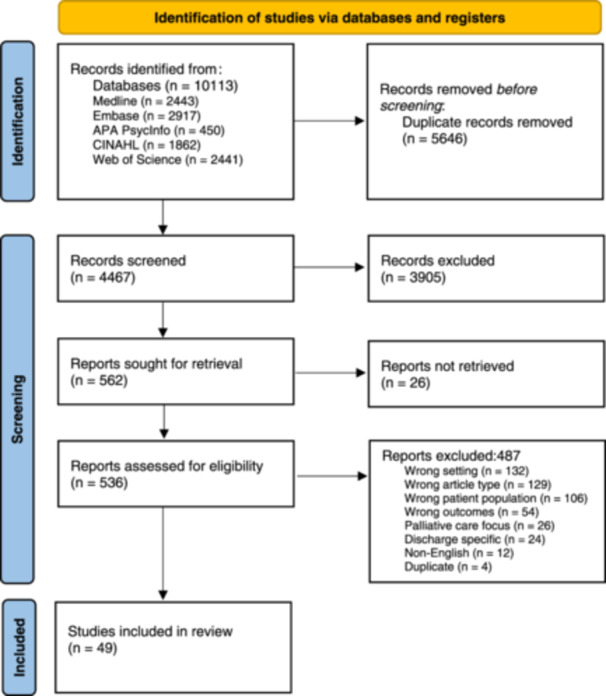
Flow chart of record search and selection process.

In total, 10,113 records were identified of which 5646 were duplicates. Title and abstract screening excluded 3905 records and 562 were reviewed in full text. Of those, 49 met inclusion criteria and form the basis for this review (Figure 1). Screening of reference lists of the included studies did not reveal any additional articles. All 49 articles included findings related to the concept of ‘user’ experience of care (Table 1); these results were included in the synthesis later.

**Table 1:** Included studies.

Author, date, country	Purpose	Sample with participant characteristics	Method/study design	Concepts	Measurement tools/outcome measures	Main results
Alves, Amendoeira, and Charepe [40], Portugal	To understand how the care partnership was experienced by parents of children with special healthcare needs	10 mothers of children with special healthcare needs	Qualitative (descriptive)	Care partnership between parents and nursing team	Non‐structured interviews (ethnobiographic orientation interviews)	The partnership was a resource for the mothers who work together with the nurses to obtain gains in well‐being, quality of life and integral development of the children. The children are at the centre of the partnership
Atout, Hemingway, and Seymour [41], Jordan	To explore the experience of decision‐making in the care of children with palliative care needs in Jordan from the perspective of their mothers	24 mothers of children with myelomeningocele, nephrotic syndrome, end‐stage renal disease, cerebral palsy, tetralogy of fallot, and hydrocephalus. Along with 12 physicians and 20 nurses	Qualitative case study approach	Decision‐making for children with palliative care	Participant observation (156 observational hours) Semi‐structured interviews	Mothers preferred to give the doctor the major role in deciding treatment. Mothers had little confidence in their ability to decide the best course and were fearful of making wrong decisions. Mothers also anticipated guilt related to decisions about their child. Sometimes mothers perceived a lack of good options impacting their decision
Baird et al. [42], United States	To understand interactions, processes and the creation of meaning for the parents of children with complex chronic conditions (CCC) and nurses in the PICU	7 parents of children with CCC (5 mothers, 2 fathers) 12 nurses	Qualitative (grounded theory)	Patient‐ and family‐centred care	Participant observation. Parent interviews Healthcare provider interviews (parent‐identified)	Identified the existence of explicit and implicit rules in a paediatric intensive care unit, all of which negatively affected the family's ability to receive care that was attentive to their needs
Baird et al. [43], United States	To explore the delivery of continuity of nursing care in the PICU from the perspective of both parents and nurses	7 parents of children with complex medical conditions admitted to the PICU 12 PICU nurses	Qualitative (grounded theory)	Continuity of nursing care in PICU	Participant observation Parent interviews Nurse interviews	Continuity for PICU patients and families was inconsistently achieved and that there was confusion among both parents and nurses about the continuity process. Both parents and nurses valued continuity. Nurses identified contextual and personal challenges to continuity
Bogetz et al. [44], United States	To examine aspects important to developing therapeutic alliance between clinicians and parents of children with severe neurologic impairment (SNI)	25 parents (17 mothers) of children with SNI 25 clinicians	Qualitative (thematic analysis)	Development of therapeutic alliance between clinicians, parents and children	Semi‐structured interviews. Survey of parents, patients and clinicians	Three themes to build therapeutic alliance including: Foundational factors must exist to establish rapport; structural factors (perspective taking and seeing the big picture) provide awareness of the parent/child experience; weathering factors (flexibility, open‐mindedness, compassion, humility) comprise the protection, security, and additional support during hard or uncertain times
Borgioli and Kennedy [45], United States	To study the causes, educational continuity and parental perceptions associated with students with multiple disabilities transitioning from school to hospital	Parents of 19 students with multiple disabilities	Qualitative (thematic analysis)	Impact on education of transitioning from school to hospital	Interviews	Only 1 in 46 hospitalizations had an educational plan. Some parents were concerned about the absence of educational services and attempted to improve service delivery. Another group of parents were not concerned regarding the absence of educational services, and noted the severity of their child's disability as the reason
Borschuk et al. [74], United States	To describe a novel behavioural health programme and examine its impact on family caregiver engagement and psychological distress on a paediatric inpatient chronic ventilator unit	53 retrospective cases and 25 parents followed prospectively	Quantitative, post‐intervention, cross‐sectional study with historical control	Family caregiver engagement Psychological distress	Qualitative caregiver engagement scale (QCES) NIH toolbox PROMIS measure, perceived stress questionnaire (PSQ)	No significant difference in family caregiver engagement pre/post intervention. There was a significant increase in caregiver participation in medical rounds from 29.7% to 66.3%. Caregivers were rated as more engaged by staff and were more likely to complete education more quickly after the intervention. Caregivers experienced a decrease in level of distress after completing a course of psychotherapy
Brady et al. [80], United States	To develop a comprehensive understanding of how families identify and communicate their child's deteriorating health with the hospital‐based healthcare team	28 parents (21 mothers, 2 fathers, 1 stepmother and 2 mother–father dyads.	Qualitative (thematic analysis)	Family identification and communication of their child's deteriorating health	Semi‐structured interviews and paper journaling.	Themes included: (1) parents are best able to understand the child's baseline (often misunderstood by HCPs); (2) there are informal/learned pathways to navigate the complex and confusing system; (3) parental advocacy and persistence are important; (4) parents are not ‘typical’ parents in that they have different roles and expertise; (5) medical culture does not consistently support partnership; (6) parents are often ‘running on empty’ due to stress, fear, lack of sleep and loss of control in hospital
Bravo et al. [85], United States	To evaluate parent–child perceptions of self‐management, self‐efficacy and health‐related quality of life (HRQol) in children with chronic illness and medical complexity (CIMC)	32 parent–child pairs	Observational, quantitative, cross‐sectional study	Self‐management Self‐efficacy HRQoL	Parent Activation Measure Self‐Efficacy Scale Acute Care Pediatric Quality of Life	Parents (56.3%) and children (40.6%) reported moderate levels of self‐management. HRQOL was correlated with both self‐management and self‐efficacy. At least 25% to 50% reported low PedsQL subscale scores, which indicate problems with physical, emotional, social, and mental domains
Bull and Gillies [73], United Kingdom	To explore the views of hospitalized school‐aged children with complex healthcare needs related to spiritual care	5 hospitalized children	Qualitative (grounded theory)	Spiritual needs of school‐aged, hospitalized children with complex healthcare needs	Semi‐structured interviews supported by photographs	Main themes: (1) The role of the child's relationships with family, friends and HCPs; (2) the impact of the hospital environment; (3) coping with invasive procedures; (4) children's views on their health and belief system
de Souza Esteves et al. [83], Brazil	To identify concerns of family members of Children with Special Health Care Needs (CSHCN) as far as care related to using technology, and to discuss nurses’ performance in the face of these concerns	6 parents or caregivers participating in the care of CSHCN with technological care demands	Qualitative (descriptive)	Health communication Concerns about technology dependence for children	Semi‐structured interviews	Concerns were distributed in a timeframe, divided between those occurring the moment the family members received the information about the technological device needed, then those which arose while accompanying the child during hospitalization, and finally those that remained after the hospital discharge
Engler et al. [81], Germany	To explore how parents of children and adolescents with life‐limiting conditions think about the hospital as place of care	13 parents (9 mothers, 4 fathers) of children with life‐limiting conditions receiving or having received specialized outpatient palliative care	Qualitative (grounded theory)	Parents' perceptions of the hospital as place of care	Unstructured narrative interviews	Parents reported feelings of vulnerability, heteronomy, and disablement associated with hospital care and were afraid that their children's needs were not adequately addressed. These perceptions resulted from hospitals' standardized care structures and over‐ and undertreatment, a lack of continuity of care, hospital pathogens, a lack of a palliative mindset, insensitive hospital staff, the exclusion of parents from the treatment and parental care of their children, the hospital stay as a permanent state of emergency, and a waste of limited life time
Giambra et al. [46], United States	To expand understanding of the process of communication between parents of hospitalized technology‐dependent children and their nurses originally detailed in the Theory of Shared Communication (TSC)	5 parents of technology‐dependent children along with 9 nurses	Qualitative (grounded theory)	Parent–nurse communication	Parent journals Semi‐structured interviews for parents and nurses	The propositions of the TSC were verified. Respect for one's own expertise and for the expertise of the other is needed to achieve mutual understanding. The 6 categories of the TSC are interrelated and each is essential to the communication process
Giambra et al. [47], United States	To determine the process of parent–nurse communication from the perspective of the parents of technology‐dependent children who have been hospitalized	*n* = 11 parents (9 mothers, 1 grandmother, 1 adoptive mother)	Qualitative (grounded theory)	Parent–nurse communication	Semi‐structured interviews	The Theory of Shared Communication was the result of this study and includes questioning, listening, explaining, advocating, verifying understanding and negotiating roles to achieve the outcome of mutual understanding of the child's plan of care
Giambra, Stiffler, and Broome [78], United States	Review evidence in literature of communication behaviours, components, concepts or processes that improve mutual understanding between nurses and parents of hospitalized technology‐dependent children to provide optimal care	6 articles	Integrative review of qualitative and quantitative studies	Communication	N/A	Providing clear information, involving parents in care decisions, trust and respect for each other's expertise, caring attitudes, advocacy, and role negotiation are important factors in shared parent–nurses communication
Grandjean et al. [48], Switzerland	To explore the specific PICU‐related sources of stress, family functioning and needs of families of chronically critically ill (CCI) patients during a PICU hospitalization	31 interviews (12 mothers, 8 fathers, 11 mother–father dyads)	Qualitative (descriptive, content analysis)	PICU‐related sources of stress Family functioning Family needs Perceived child's quality of life	Semi‐structured interviews	Five themes: (1) high emotional intensity, (2) PICU‐related sources of stress, (3) evolving family needs, (4) multifaceted family functioning, and (5) implemented coping strategies
Guerini et al. [72], Brazil	To identify the perceptions of relatives taking care of technology‐dependent children regarding the stressors that affect their relationships	9 caregivers (8 mothers, 1 sister)	Qualitative	Stressors related to providing care to technology‐dependent children	Semi‐structured interviews	Three categories emerged: ‘I live for him/her now’; ‘Stressful situations happen all the time’; The man × woman relationship changed. Women identified the occurrence of multidimensional, everyday changes in their family life and, particularly, in their personal life, as they practically took over the whole care alone, abandoned their job, leisure, and ‘being a woman’
Hagvall, Ehnfors, and Anderzén‐Carlsson [82], Sweden	To describe parental experiences of caring for their child with medical complexity during hospitalization for acute deterioration	9 parents (7 mother, 2 fathers)	Qualitative (descriptive)	Parental needs Attitudes of staff	Semi‐structured interviews	The main theme was a balancing act between acting as a caregiver and being in need of care. Parents felt they were in a vulnerable situation. They were acting as the child's ambassador, wanting to be involved in the care, ensure their child is treated well and have staff respect their knowledge
Henderson et al. [76], United States	To describe children's experiences with PICU care for paediatric chronic critical illness (CCI)	7 parents along with 21 physicians, 15 nurses/nurse practitioners, 4 social workers and 1 other	Qualitative	Children's experiences of care in the PICU	Semi‐structured interviews	Themes reflected the different perspectives. For the child, living in the PICU with acute care models a poor match for needs. For families, there were acute‐on‐chronic stressors and barriers to visitation. Clinicians found it difficult to meet developmental needs and manage an altered clinician‐parent dynamic. It was also difficult to establish ‘PICU’ goals of care and envision a transition to home
Hoang et al. [49], United States	To understand the current practice of goal setting at the beginning of hospitalization by exploring the perspectives of parents of hospitalized children and their hospital physicians	27 parents/legal guardians (15 mothers, 11 fathers, 1 guardian) along with 16 physicians. Half of the parents had children with complex, chronic conditions and half did not	Qualitative (modified grounded theory)	Goal setting during hospitalizations	Semi‐structured interviews with parents and their child's attending physicians.	(1) Majority of hospitalized children's parents want to share their goals with physicians. (2) Parents and physicians share the same underlying goal of getting the child better to go home. (3) Parents of children with chronic diseases identified nonhospital goals that were not addressed. (4) Physicians do not explicitly elicit but rather assume what parents’ goals of care are. (5) Factors related to patient, parent, and physician were identified as barriers to goal setting
Hoang et al. [50], United States	To explore the perspectives of parents of hospitalized children and their hospital providers on facilitators and barriers to shared decision‐making (SDM) in the hospital and identify strategies to increase SDM	27 parents/legal guardians (15 mothers, 11 fathers, 1 guardian) along with 16 physicians. Half of the parents had children with complex, chronic conditions and half did not	Qualitative	Shared decision‐making	Semi‐structured interviews	Four themes emerged: (1) parents and providers value different components of SDM; (2) providers assume SDM is easier with parents of children with medical complexity; (3) factors related to providers, parents, patients, and family‐centred rounds were identified as barriers to SDM; and (4) parents and providers identified strategies to facilitate SDM in the hospital
Iannelli et al. [18], Australia	To examine levels of parental satisfaction with inpatient care for children with cerebral palsy (CP) at a tertiary care hospital and identify areas for improvement	130 parents (90 without disabilities, 40 with CP)	Observational, cross‐sectional quantitative study	Parental satisfaction with inpatient care	Questionnaire developed by the research team assessing six areas of the hospital stay: the admissions process, my child's personal care, my child's medical care, overall care of the child, my experience in the hospital and keeping up to date in the hospital.	Parents of children with CP were significantly less satisfied than parents of children without a disability in particular for the child's personal care, the child's medical care, overall care and experience in hospital
Kemp et al. [24], Canada	To examine the comprehensive inpatient experience of children with medical complexity (CMC) by using a validated patient‐reported experience measure and compare the results with all other respondents at 2 academic paediatric hospitals in a western Canadian province	4197 parental caregivers (1515 parents of CMC; 2682 parents of non‐CMC)	Observational, cross‐sectional quantitative study	Inpatient experience of CMC	The Child Hospital Consumer Assessment of Healthcare Providers and Systems survey. (Child HCAHPS)	When compared with the non‐CMC cohort, a lower percentage of parents of CMC reported top box scores on 13 of the 18 standard Child HCAHPS measures. The largest differences observed were for quietness of hospital room at night, responsiveness to the call button, staff paying attention to the child's pain, and communication between parents and nurses. Additionally, CMC had lower results on 7 of 10 stand‐alone organization‐specific items, especially for providers having a clear understanding of the child's condition, the overall rating of care from nurses, and providers doing everything they could to help with pain
Leary et al. [51], United States	To elicit parent perspectives on circumstance surrounding 30‐day readmissions for children with medical complexity (CMC)	20 parents	Qualitative (modified grounded theory)	Parent perceptions of CMC hospital readmissions	Semi‐structured interviews	The majority of parents did not identify any factors that contributed to readmission. Some parents felt perceived challenges associated with chronicity of care and transitions of care that might influence readmissions, including frequency of hospital use, symptom confusion, lack of inpatient continuity, resources needed but not received, and difficulty filling prescriptions
LeGrow et al. [52], Canada	To test the feasibility of a parent‐briefing intervention for parents of hospitalized children with complex healthcare needs	31 parents along with 18 physicians and 25 nurses	Post‐intervention, cross‐sectional study	Feasibility and protocol compliance of a parent‐briefing intervention	Feasibility questionnaires developed by the research team Checklist to monitor protocol compliance.	Sixty‐eight briefings were carried out. Parents and nurses evaluated the briefings in a favourable manner, whereas physicians' ratings were mixed
Lin et al. [69], United States	To describe parent perspectives of shared decision‐making (SDM) for children with medical complexity (CMC) and identify opportunities to improve elements of SDM specific to this vulnerable population	32 parents (27 mothers, 5 fathers)	Qualitative (modified grounded theory)	Shared decision‐making	Semi‐structured interviews.	Three categories of themes emerged: participant, knowledge, and context. Key opportunities to improve SDM included: providing a shared decision timeline, purposefully integrating patient preferences and values, and addressing uncertainty in decisions
Madrigal et al. [53], United States	To assess sources of support and guidance on which parents rely when making difficult decisions in the paediatric intensive care unit and to evaluate associations with anxiety, depression and positive and negative affect	86 parents (60 mothers, 26 fathers)	Observation, quantitative, prospective study	Sources of support and guidance Anxiety, depression and positive and negative affect	Instrument developed by research team—‘Sources of Support and Guidance’ The Positive and Negative Affect Scale (PANAS) The Hospital Anxiety and Depression Scale (HADS), the Adult Dispositional Hope Scale,	Parents reported doctors, nurses, friends, extended family, and instinct as the strongest sources of support and guidance when making difficult medical decisions. Support groups, spiritual leaders, and church community ranked lowest
Madrigal et al. [54], United States	To assess decision‐making preferences of parents in the paediatric intensive care unit and test whether preferences differed with demographics, complex chronic conditions, prior admissions and parental positive and negative emotional affect	86 parents (60 mothers, 26 fathers)	Observation, quantitative, prospective study	Parental and shared decision‐making Parental emotional affect	Instrument developed by research team—‘Parental Decisions Preference’ The Positive and Negative Affect Scale (PANAS) The Hospital Anxiety and Depression Scale (HADS), the Adult Dispositional Hope Scale	The majority preferred shared decision‐making with their doctors (40.0%) or making the final decision/mostly making the final decision on their own (41.1%). Increased levels of positive affect were associated with a higher likelihood of preferred shared decision‐making over alternative modes of decision‐making. Parents' degree of negative affect was not associated with any of the decision‐making preferences. The presence of chronic complex conditions, previous admissions to the hospital had no influence on preference for decision‐making
Mimmo, Harrison, and Hinchcliff [55], Australia	To explore the evidence of patient safety outcomes for children with intellectual disability (ID)	16 studies	Systematic review	Patient safety outcomes for children with intellectual disability	N/A	Three themes: the impact of the assumptions of healthcare workers (HCWs) about the child with ID on care quality and safety outcomes; reliance on parental presence during hospitalization as a protective factor; and the need for HCWs to possess comprehensive understanding of the IDs experienced by children in their care, to scientifically deduce how hospitalization may compromise their safety, care quality and treatment outcomes
Mimmo et al. [56], Australia	To systematically identify and synthesize peer‐reviewed qualitative evidence of the parental experience of hospitalization with a child with intellectual disability (ID)	11 articles	Systematic Review	Parents experience of hospitalization with a child with intellectual disability	N/A	Five themes were identified: (a) being more than a parent, (b) importance of role negotiation, (c) building trust and relationships, (d) the cumulative effect of previous experiences of hospitalization and (e) knowing the child as an individual
Murrell et al. [57], United States	To understand parents' perspectives of their care experiences in emergency, hospital and clinical care settings to identify gaps in care	29 Parents of children with Spinal Muscular Atrophy (SMA) (18 mothers, 11 fathers)	Qualitative	Experience of care	Semi‐structured interviews	Three overarching themes emerged from parent interviews describing a range of experiences surrounding diagnosis, informed medical decision‐making and acute care practice
Nolan et al. [58], United States	To describe and compare perceptions of parents (during their child's hospitalization) with those of paediatric interns and paediatric hospitalists of long‐term health‐related quality of life (HRQoL) of children with severe disabilities	40 parents along with 22 hospitalists and 20 paediatric interns	Mixed methods, observational, cross‐sectional study	Perceptions of parents and physicians on long‐term health‐related quality of life (HRQoL)	KIDSCREEN‐10 Instrument developed by research team Semi‐structured interviews	Parents rated their child's HRQoL higher than physicians. Parents and physicians also expressed different goals for treatment. Parents expressed optimism despite uncertainty regarding their child's future, whereas physicians anticipated increased medical complications and focused on caregiver burden
Noyes [59], United Kingdom	To describe the views and experiences of young, ‘ventilator‐dependent’ people (and their parents) regarding the care and services they received, and to find out whether their needs were met during prolonged periods in intensive care units	18 children or youth (8 girls, 10 boys) and 25 family members	Qualitative (phenomenology)	Meeting health needs, social needs environmental needs, and aspirations for the future	Focused interviews, some with draw/play techniques	Living in an intensive care unit is inappropriate and detrimental to health and well‐being of children. Four themes included: (1) The experience of being dependent on a ventilator; (2) The psychosocial impact of the intensive care unit; (3) Play and leisure; (4) Rehabilitation
Oulton, Sell, and Gibson [60], United Kingdom	To identify what parents want from their relationship with healthcare professionals	9 parents of children and young people (CYP) with intellectual disability (ID)	Qualitative (ethnography)	Relationship between parents and healthcare professionals	In‐depth interviews Participant observation	The overriding requirement was the need for a genuine partnership with professionals. Seven elements ideally characterize this partnership: Preparation, Accessibility, Reliability, Trust, Negotiation, Expertise and Respect (PARTNER)
Oulton, Sell, and Gibson [61], United Kingdom	To understand the hospital‐related needs and experiences of children and young people (CYP) with intellectual disabilities	*n* = 9 CYP with ID	Qualitative (ethnography)	Hospital‐related needs and experiences of CYP with ID	Participant observation Tailored interviews, including art‐based research methods. Informal parent discussions	Five themes, explained what is important to CYP with intellectual disabilities in hospital: (i) little things make the biggest difference, (ii) eliminate unnecessary waiting, (iii) avoid boredom, (iv) routine and home comforts are key and (v) never assume. LEARN
Phua et al. [29], Australia	To evaluate differences in perceptions of inpatient care by parents of children with cerebral palsy (CP) compared to parents of able‐bodied children	130 parents (40 parents of children with CP, 90 parents of children without disabilities)	Observational, quantitative, cross‐sectional study	Perceptions of inpatient care Parental concern and dissatisfaction	*Perceived Stress Scale (PSS‐10)* Instrument developed by the research team asking about the admissions process, care of child in hospital, communication and confidence in doctors and nurses, participants’ personal experience in the hospital, information exchange	Overall, parents of able‐bodied children were more satisfied with the hospitalization than parents of children with CP. Significant differences were found in four of the five areas assessed: the admissions process; the care that their child received; their communication and confidence in doctors and nurses; and their personal experience of the hospital. Parents of disabled children displayed a much higher mean score on the Perceived Stress Scale, but no correlation was found between this scale and the satisfaction questionnaire for either group
Reeves, Timmons, and Dampier [62], United Kingdom	To understand the negotiation of care as experienced by the parents of technology‐dependent children in a hospital context	6 parents	Qualitative (exploratory)	Negotiation of care	Semi‐structured interviews	Parents felt that their roles as parents were not considered enough by nurses and they tended to be seen as carers, not parents. Negotiation of care was not always apparent. Instead, nurses often made assumptions about parental involvement in care. Parents wanted to carry out care when in hospital, but were not always given choices. Parents also reported a desire for more confident nurses
Rennick et al. [79], Canada	To elicit an in‐depth understanding of parents' experiences caring for children with chronic medical complexity (CMC) in the PICU	17 parents of CMC	Qualitative (interpretive description)	Parents experience of caring for CMC in the PICU	Semi‐structured interviews	Parents of CMC expected to continue providing expert care during PICU admission, but felt their knowledge and expertise were not always recognized by staff. They emphasized the importance of parent–staff partnerships. Four themes were identified: (1) ‘We know our child best’; (2) when expertise collides; (3) negotiating caregiving boundaries; and (4) the importance of being known
Seliner, Latal, and Spirig [63], Switzerland	To assess the effectiveness of a family‐centred care (FCC) intervention provided by an advanced practice nurse (APN) for parents of children with profound disabilities undergoing surgery	28 parents (23 mothers, 5 fathers), 14 in the intervention group and 14 parents in the control group	Quasi‐experimental, post‐intervention with historical control	Satisfaction with family‐centred care	Measures of Processes of Care (MPOC‐20) questionnaire Impact of Family Scale (IFS) Semi structured interviews (intervention group only)	No significant differences were found between the intervention and non‐intervention group for the MPOC‐20 domains. Three main areas of concern emerged from interviews: feeling well prepared, expecting coordinated and continuous information, and, expecting to be an equal part of the team
Seliner, Latal, and Spirig [27], Switzerland	To assess parental burden of care, satisfaction with family‐centred care, and quality of life (HRQoL) of parents and the well‐being of their hospitalized children with profound intellectual and multiple disabilities (PIMD) and determine the relationship among these factors	117 parents (98 mothers and 19 fathers) completed surveys 26 (24 mothers and 2 fathers completed interviews	Mixed methods, observational, cross‐sectional study	Parental burden of care Family‐centred care Quality of life	Impact of Family Scale (IFS) Short Form Survey (SF‐36) for HRQoL Measure of Processes of Care (MPOC‐20) DISABKIDS Smiley questionnaire Semi‐structured interviews	Parents indicated a substantial impact on burden of care and parental health‐related quality of life. Significant correlations with the hospitalized children's well‐being were for burden of care and quality of life. Qualitative results showed parents struggling to safeguard their children and worrying most about the children's well‐being
Shilling et al. [25], United Kingdom	To synthesize qualitative research reporting the experience of disabled children as hospital inpatients and identify factors which affect their care	8 articles	Systematic review of qualitative studies	Experiences of care of children in hospital	N/A	Communication between children and staff was a dominant theme and comprised giving the child information about their condition and appropriate involvement of the child/young person in discussions and decision‐making that affected them. Also, important was communication between parents and staff, particularly around the division of care for their child. Other themes included emotions, particularly fears, the ward environment and confidence in staff
So et al. [84], Canada	To examine parental perceptions and perceptions of care of CMC during prolonged hospitalization in the context of an inpatient programme [the Beanstalk Program (BP)] that strives to enhance the developmental experiences of chronically ill, long‐term hospitalized children and their families	20 parents completed the questionnaire. 11 parents participated in interviews (9 mothers and 2 fathers)	Mixed methods, post‐intervention	Parental experiences and perceptions during prolonged hospitalization	Measures of Processes of Care (MPOC‐20) questionnaire Semi structured interview	Results on the MPOC were generally positive, with Respectful and Supportive Care the highest and Providing General Information the lowest. Interview data generated key themes: (a) parents strive for positive and normal experiences for their child within the hospital environment; (b) parents value the focus on child development in the midst of their child's complex medical care; and (c) appropriate developmentally focused education helps parents shift from feeling overwhelmed with a medically ill child to instilling feelings of confidence and empowerment
Sobush [64], United States	To provide a description of a CMC programme model	Unknown	Descriptive	Description of a care programme for CMC. Informal feedback	Not indicated	Lessons learned: − Multiyear strategic planning was required to become operational − A physician and advanced practice nurse dyad assigned to each patient reduced miscommunication about clinical care planning − Dynamic individual healthcare plans can be translated into the electronic medical record and shared with community providers − Patient enrolment growth forecasts were underestimated
Stone et al. [65], United States	To compare parental perceptions of inpatient family‐centred care for children with complex chronic medical illnesses (CCMI) in structured clinical programmes (SCPs) with those who are not in SCPs	214 parents (98 parents of children in SCPs, 116 parents of children not in SCPs).	Observational, quantitative, cross‐sectional study	Family‐centred care	Measures of processes of care (MPOC)−56	Parents of children cared for in SCPs reported a higher scale for the provision of general information and providing respectful and supportive care compared to parents of children in non‐SCPs. There were no differences in the other scaled scores between the two study groups
Taib, Beng, and Chan [66], Malaysia	To explore the challenges faced by parents with children who have complex neurological conditions, their coping strategies, needs and expectations	11 parents (4 mothers, 7 fathers)	Qualitative (grounded theory)	Challenges faced by parents Coping strategies, needs and expectations	Semi‐structured interviews	8 challenges: physical wellbeing, environment, relationship, financial, occupational, rational, mental and spiritual. Coping strategies comprised problem‐focused issues related to the key challenges in the caregivers' context
White et al. [7], United States	To describe the development and implementation of a new care model for hospitalized children with medical complexity and summarize feedback from key stakeholders, including trainees, providers, nurses and families	Unknown	Descriptive	Description of a care programme for CMC. Informal feedback	Interviews with families HCP surveys	Families and providers noted improvements in care coordination with the new care model. Remaining challenges include balancing resident autonomy and attending supervision, as well as supporting providers in delivering care that can be emotionally challenging
Williams et al. [75], United States	To obtain feedback on communication, care coordination and transitions in care for hospitalized children with medical complexity (CMC).	6 parents along with 15 nurses and 9 other HCPs	Mixed methods, post‐intervention	Communication Care coordination Transitions in care	Instrument developed by the research team including forced choice and open‐ended questions	Parents' ratings of communication, care coordination and transitions in care were generally high. Transitions from other facilities to the emergency department and unit received lower ratings
Woodson et al. [67], United States	To assess the impact of specific child, parent and family factors contributing to family hardiness	68 parents (43 mothers, 16 fathers and 9 guardians)	Mixed methods, observational, cross‐sectional	Family hardiness and resiliency	Family Hardiness Scale Semi‐structured interviews	Two predictors demonstrated significant effects for hardiness: age in years and the number of negative coping strategies for the child. Four themes represent positive child coping: (1) staying active to distract from stressors; (2) physical comfort from a loved one; (3) keeping a positive attitude; and (4) the child being able to participate in therapy at the hospital that speeded child recovery. Three themes representing negative child coping: (1) being noncompliant about using medical equipment; (2) strong negative emotional reactions; and (3) a lack of knowledge about his/her illness
Wright‐Sexton et al. [68], United States	To describe the experiences of parents and providers of children with chronic critical illness (CCI) specifically around isolation during PICU admission	12 parents (9 mothers, 3 fathers) of children with CCI along with 7 PICU physicians and 8 nurse practitioners	Mixed methods, observational, cross‐sectional study	Isolation during PICU admission	Semi‐structured interviews Center for the Epidemiological Studies of Depression Short Form (CES‐D 10) Medical Outcomes Study Social Support Survey (MOS‐SS)	Parents did not feel medically isolated, although providers did. Parents self‐reported adequate social supports but scored high on depression scales suggesting a disconnect between perceived and actual support

Abbreviations: CMC, children with medical complexity; HCPs, healthcare providers; PICU, paediatric intensive care unit.

Table 2 presents an overview of included studies. The majority of existing evidence comes from the United States (*n* = 25), the United Kingdom (*n* = 5) or Canada (*n* = 4). Most studies enroled CMC (*n* = 34) or children with severe disabilities (*n* = 10) as non‐diagnostic study populations with the remainder focused on children with cerebral palsy (*n* = 2), spinal muscular atrophy (*n* = 1) or ventilator dependence (*n* = 2). Only a small number of studies enroled CMC themselves, with mothers forming a large majority of the study populations. The studies used predominantly qualitative methodologies (*n* = 27) with a lesser proportion using quantitative (*n* = 10) or mixed (*n* = 6) approaches (Table 3). The results of the thematic analysis will be described, followed by an overview of the study methodologies.

**Table 2:** Overview of included studies.

Characteristics	No. of studies	Studies
Country		
Unites States	25	[7, 38, 39, 40, 41, 47, 49, 50, 51, 52, 53, 54, 55, 56, 60, 62, 65, 66, 68, 69, 70, 71, 72, 73, 74]
United Kingdom	5	[25, 45, 58, 75, 76, 77]
Canada	4	[24, 57, 59, 78]
Brazil	2	[43, 63]
Germany	1	[79]
Australia	4	[18, 29, 80, 81]
Portugal	1	[37]
Jordan	1	[82]
Switzerland	3	[27, 46, 83]
Sweden	1	[48]
Malaysia	1	[42]
Setting		
PICU	9	[50, 54, 62, 65, 66, 72, 75, 78, 83]
Inpatient unit	21	[7, 18, 27, 29, 40, 41, 43, 46, 47, 48, 51, 55, 56, 57, 58, 59, 69, 73, 74, 76, 77]
Both	3	[24, 68, 70]
Other/not specified^a^	16	[25, 37, 38, 39, 42, 45, 49, 52, 53, 60, 63, 71, 79, 80, 81, 82]
Participants		
Parental caregivers	41	[7, 18, 24, 27, 29, 37, 38, 39, 40, 42, 43, 45, 46, 47, 48, 49, 50, 51, 52, 53, 54, 55, 56, 57, 58, 59, 60, 62, 63, 65, 66, 68, 69, 70, 71, 72, 73, 78, 79, 82, 83]
CMC	3	[41, 76, 77]
Both	2	[74, 75]
Other/not specified^a^	3	[25, 38, 80]
Sample size		
5–10	13	[37, 40, 43, 45, 48, 51, 58, 62, 63, 65, 72, 76, 77]
11–24	10	[39, 42, 54, 57, 60, 71, 78, 79, 82, 83]
25–100	16	[18, 41, 46, 47, 49, 50, 52, 55, 56, 59, 66, 68, 69, 73, 74, 75]
> 100	4	[24, 27, 29, 70]
Other/not specified^a^	6	[7, 25, 38, 53, 80, 81]

a Includes review articles.

**Table 3:** Study design.

Study design	No. of studies	Studies
Qualitative	27	
Grounded theory	10	[39, 40, 42, 55, 56, 65, 71, 72, 76, 79]
Unspecified	5	[37, 49, 62, 69, 82]
Descriptive	4	[43, 48, 63, 83]
Thematic analysis	3	[47, 60, 68]
Ethnography	2	[58, 77]
Phenomenology	2	[45, 75]
Interpretive description	1	[78]
Quantitative	10	
Observational, cross‐sectional study with control group	4	[18, 24, 29, 70]
Observational, cross‐sectional study with no control group	2	[52, 74]
Post‐intervention, cross‐sectional study with no control	1	[59]
Post‐intervention, cross‐sectional study with historical control	1	[73]
Observational, prospective study	2	[50, 66]
Mixed methods	6	
Observational, cross‐sectional study	4	[27, 41, 51, 52, 54]
Quasi‐experimental, post‐intervention with historical control	1	[46]
Post‐intervention, no control	1	[57]
Descriptive	2	[7, 53]
Review	4	[25, 38, 80, 81]

### 3.1 Part 1: Results of Thematic Analysis

There were seven themes identified that described essential components of inpatient experience of care from the perspective of CMC and their families. These include the following: (1) negotiation of care roles; (2) shared decision‐making (SDM); (3) common goal setting for the hospitalization; (4) *relationships* between CMC, their families and HCPs; (5) integration of *expertise of CMC, their families and HCP*; (6) communication strategies and practices; and (7) the hospital setting and environment itself.

#### 3.1.1 Negotiation of Care Roles

Care roles must be negotiated and established at different stages during the course of inpatient care, which contributed to a sense of partnership and teamwork [25, 40, 46]. An open discussion about roles and responsibilities for both nurses and parents promoted confidence and mutual understanding [78]. Negotiation of care boundaries took time and work to establish [79, 80], with occasional tension due to a lack of role clarity [63, 80]. Some parents found that they needed to fight with HCPs to enact their roles and provide the parental care their child required [79, 81], although this was not a universal finding [55]. At times, negotiation around a parent's readiness to learn about care interventions and technologies was also required [40, 83]. Authors of one review article suggested that adjustments in parental roles in hospital were essential to ensure safe and high‐quality care, particularly for children with intellectual disabilities [55].

#### 3.1.2 Shared Decision‐Making

Many studies described how parents want to partner in making decisions regarding their child's care [27, 46, 50, 56, 63, 78, 80]. Parents valued being involved in decision‐making [48] or in control of decision‐making [69]. Many expected to be an equal part of the team [63] and wanted to be involved in choices regarding their child's treatments [54, 82]. It was important to parents that decisions were based on their individual child as opposed to the diagnosis [57]. Children also expressed their desire to be involved in decision‐making [25]. SDM was enabled by good communication skills and active listening (particularly to family values and concerns) [50, 57] as well as parental knowledge and sources of information [69]. Barriers to SDM included poor HCP communication skills, a large medical team that could be intimidating, insufficient time for parents to prepare and health system factors (e.g., availability of specific HCPs) [50]. The decision time horizon (how quickly the decision needed to be made) influenced parental participation in SDM [57, 69]. Even though they desired high degrees of involvement, parents frequently expressed uncertainly around decision‐making, which was under‐addressed [69]. Quantitative studies reinforced that many parents desire SDM (40%), but a significant proportion also wanted to make the final decision largely on their own (41%) [54]. In only one study, conducted in the Middle East, did participants (mothers) feel that decision‐making should be the responsibility of the doctor [41].

#### 3.1.3 Common Goal Setting

Closely related to specific, contextual SDM was setting common goals for the hospitalization. Physicians typically did not elicit parents' goals for the child at the beginning of hospitalization; however, many parents wanted to share these goals with physicians [49]. Parents described setting these larger goals for their child or for the hospital admission as an important part of their role [84]. Typically, physicians and parents shared the underlying goal of getting the child better to go home [49]. Incongruency of other goals between parents and physicians also existed [49, 50, 55, 58, 68, 80]. Goals that parents felt were not addressed included limiting unnecessary testing, establishing follow‐up plans, understanding their child's medical diagnosis and prognosis, developing strategies to prevent future hospitalizations, improving their child's daily life, creating palliative care plans and feeling empowered as a parent [49, 50, 81]. In one study, researchers found that parents were more likely to have goals related to their child's life skills such as becoming more independent and learning to communicate, whereas physician goals were more likely to focus on decreasing parent burden, keeping the child out of hospital, avoiding complications and coordinating care [41].

#### 3.1.4 Relationships Between CMC, Families and HCPs

Relationships with HCPs, as described by CMC and their families, were grounded in rapport, continuity of care and trust [70]. A recent review by English et al. proposed the following definition of rapport in the healthcare encounter: ‘a perception of connection with another individual based on respect, acceptance, empathy and a mutual commitment to the relationship’ [70]. Parents included in this review were able to identify HCP factors that contributed to rapport including trustworthiness, transparency and respect along with supportive knowledge and actions [40, 44, 60]. The HCP's ability to see the situation from the parent's perspective and look at the big picture facilitated rapport, as did a lack of bias about the child [44]. Rapport was maintained through difficult situations when HCPs were flexible, open‐minded, compassionate and humble [44]. Rapport was inhibited when HCPs misunderstood a child's baseline (in relation to their current state) or failed to understand or appreciate parental intuition [80]. Insensitive staff [81] and medical culture, including the fear of being labelled a ‘difficult’ parent [80], were also barriers to building rapport. Some quantitative studies revealed that parents of CMC report lower ratings of staff awareness of their child's needs compared to parents of non‐complex children [29]; however, they also tend to indicate that ‘respectful and supportive care’ as one of their most positive experiences [27, 65, 84].

Definitions of continuity of care in the literature tend to emphasize two main components: a continuous caring relationship and seamless coordination across providers/systems [71]. Parents in this review emphasized the importance of both components in their hospital experiences [27, 43, 44, 45, 56, 79, 82] and the negative impacts when continuity was lacking [43, 48, 51, 56, 81]. Continuity had a positive impact on quality of care [43, 56] and fostering individualized care [43]. CMC themselves valued continuous relationships and specifically noted that this would facilitate comfort and support [25]. A lack of continuity negatively impacted care experiences regarding coordinated treatment plans, patient management [43, 48, 56, 81] and readmissions [51]. Without continuity, parents were concerned that HCPs would not have adequate knowledge of the child to provide competent care [43]. Parents identified breakdowns in continuity of care and resulting poor experiences that influenced their expectations of future hospitalizations [56]. Continuity was viewed as the foundation for rapport [44], trust and a sense of security with HCPs [27, 43, 79, 82], as well as creating secure attachments between nurses and patients/families [43, 62].

Trust was central to the relationship between CMC/family and HCP [44, 47]. Trust was built through shared experiences with HCPs throughout the child's illness [80], engaging in high‐quality communication [48], continuity [27, 43, 56], working in partnership [56] and observation of healthcare teams working well together [80]. Giambra, Stiffler, and Broome found that trust was the most important need perceived by parents [78]. Some parents described feeling a lack of trust in HCPs [47, 60, 82]. When trust was not established, parents could be reluctant to leave their child's bedside [82]. Negative past experiences with hospitalizations could adversely impact the formation of trust in future relationships and encounters [47].

#### 3.1.5 Integration of Expertise

The multiple forms of expertise were highlighted in many of these studies. In addition to professional and technical expertise of HCPs, there was also parent expertise based on knowledge acquisition, familiarity with the child and intuition.

Parent expertise was acquired through caring for their complex child at home, skill development, independent enquiry and connecting with other parents [43, 72]. Partnership was facilitated when parents' individualized knowledge and expertise about their child was respected and incorporated into decision‐making and provision of care [60, 78, 80]. Parents stated that HCPs need to listen to families as experts in their child's care [60, 62, 75] and parents expressed dissatisfaction when nurses wanted to control how care was provided [62]. Some parents felt that their relationship with the medical team should differ from other parents of non‐CMC due to their unique expertise around their complex child [68].

Parents did not always perceive that their expertise was valued, which led to poorer hospital experiences [79]. Some parents felt excluded from provision of care, prevented from fulfilling their roles as parents [79, 81] and unappreciated for the care that they provided for their child [51]. Parents felt their presence as experts in their child's care was a protective factor in ensuring high‐quality care and preventing errors [55]. In one study, researchers found that the sophisticated knowledge of some families could significantly alter the care dynamic [76].

Parents described the importance of HCP expertise for the development of optimal partnerships. They wanted HCPs to demonstrate specific training and knowledge in their child's condition [25, 60], special care competencies, managing complex medical situations [27], knowledge of various intellectual disability labels [55] and advocacy [78]. HCPs and parents were able to develop partnerships when HCP expertise was present and confidently enacted [25, 51, 56, 60]. Parents reported that a lack of HCP knowledge, which led to incorrect assumptions about CMC, could result in poor‐quality care [55]. In one study where children were interviewed, children expressed that they wanted their doctors and nurses to display competence, knowledge and organization [25].

#### 3.1.6 Communication Practices

An additional focus in existing research relates to the practical aspects of communication, regardless of the nature of the HCP relationship and presence/absence of continuity.

The ability to share information clearly and accurately was an important contributor to partnership [45, 48, 57, 63, 75, 78, 80]. Parents wanted to be kept up to date with a plan of care [75, 78, 80], daily goals and test results [75]. In some cases, parents wanted additional information and discussion to clarify risks and benefits of treatment options [57]. Communication deficiencies were evident in the poor integration of home care routines and schedules in hospital care, which resulted in errors [80] and complications at the time of discharge [75]. Parents felt that improved information sharing could smooth transition for school services when in hospital [45]. In quantitative studies, parents of CMC rate ‘sharing general information’ among the lowest levels of satisfaction, significantly lower than parents of non‐CMC [27, 65, 84].

Various strategies were described that enhanced the quality of communication [80]. Parents valued having HCPs who were accessible [60, 78], reliable to answer questions [60], engaged in frequent face‐to‐face discussions [80] and demonstrated good listening skills [46, 48, 78]. Words of compassion and kindness benefited parent–nurse communication [78]. Children valued nurses who explained painful procedures and used humour to decrease stress [25]. Giambra et al. described the Theory of Shared Communication (TSC) [46, 47], outlining the need to respect one's own and each other's expertise alongside critical communication behaviours including acts (questioning and listening), functions (explaining and advocating) and outcomes (verifying understanding and negotiating roles) [46, 47].

Several negative communication approaches were described, including ‘rude’ or abrupt manners [60]. Conflicting information provided by different HCPs was perceived as poor communication [75], as was disclosing a diagnosis without adequate expressions of empathy or support [57]. Assumptions, stereotypes and judgemental language about the child consistently led to a lack of effective communication [55].

#### 3.1.7 Hospital Setting and Environment

Parents described in detail how routine disruption impacted their experience of care [65]. Typical hospital procedures, such as frequent vital sign checks, disrupted the child's routine and negatively impacted the experience of care [51, 57, 76]. Baird et al. described implicit and explicit rules within the PICU [43]. Parents were implicitly expected to know the working hours of nursing staff, the educational needs of the physicians in training, and set delivery times for supplies and medications [42]. Parent requests to vary or interrupt this schedule were not welcome [42]. Rules could be bent to provide personalized care but this also caused confusion when rules were adjusted for some families but not others [42]. Adapting hospital routines to the needs of individual CMC was viewed as positively impacting the experience of care [60].

The physical and social environment in the hospital impacted the experience of children and families. The environment could undermine relationships between parents and children due to minimal privacy, a lack of control and a loss of personal identity [25, 59, 62]. Hospitals caused disruption to sleep [24, 60, 66, 73], were noisy [24, 60, 73] and placed children at risk of nosocomial infection [66, 81]. The introduction of medical technologies in the child's care caused anxiety [83]. Parents identified challenges associated with ‘living in hospital’ including access to appropriate accommodations for themselves [48]. The need for adequate sleep conflicted with the parent's need to stay at their child's bedside to ensure quality care [82].

### 3.2 Part 2: Study Methodologies

The study methodologies were qualitative (*n* = 27), quantitative (*n* = 10) and mixed methods (*n* = 6) (Table 3). Four were interventional studies [52, 63, 84] and 12 were observational [18, 24, 27, 29, 53, 54, 58, 65, 68, 75, 85]. Of the observational studies, half (*n* = 6) used a questionnaire of their own design, measuring concepts including satisfaction, feasibility, sources of parent support, decisional preferences and experiences of communication/coordination [18, 29, 52, 53, 54, 75]. Three studies used the Measures of Processes of Care (MPOC) survey [27, 65, 84], while one each used the Child Hospital Consumer Assessment of Healthcare Providers and Systems Survey (CHCAHPS) [24] and the Medical Outcomes Study Social Support Survey [68]. The MPOC and CHCAHPS both measure a broad range of experience of care, whereas the MOS Social Support survey measures an individual's sources of compassion, assistance, information and emotional support. Other studies measured health‐related quality of life using either the PedsQL [85] or the KIDSCREEN‐10 [58]. One study measured family hardiness using the Family Hardiness Scale [67].

Interventional studies that evaluated care models for CMC were described in four articles [52, 63, 74, 84]. Improving communication/coordination was a core tenet of all programmes and all were either quantitative or mixed method designs. Interventions included a behavioural health programme focusing on psychosocial support [74]; two programmes using structured communication tools [52, 63]; an inpatient programme intended to enhance developmental experiences of CMC [84]; a care coordination model for inpatient and outpatient care [64]; a single inpatient nursing unit designated for CMC; and a multifaceted, multidisciplinary HCP team [7].

Potential applications for clinical practice and healthcare delivery based on the results of this review are presented in Table 4.

**Table 4:** Potential applications for practice.

Themes	Practice implications
Negotiation of care roles	Engage in explicit and open discussion about the roles of parents and other members of the healthcare team. These can overlap and vary, both over time and with different individuals.
Shared decision‐making	Facilitate child and parent involvement in decision‐making by individualizing information, addressing uncertainty and practicing active listening.
Common goal setting	Recognize that the goals for the hospitalization may be different between children/parents and the healthcare team. Explicitly establish and agree upon goals that reflect the priorities of families and HCPs.
Relationships between CMC, families and HCPs	Optimize the provision of continuity of relationships with HCPs wherever possible. Strive to achieve optimal coordination of care and continuity of the child's health information. Personally acknowledge and address personal biases regarding CMC and their families. Recognize the complexity of establishing trust due to previous negative experiences. Recognition could include formal or informal avenues where families could share these experiences with hospitals and offer suggestions to prevent similar occurrences.
Integration of expertise	Recognize the need to integrate professional expertise with the unique expertise brought by parents of CMC. Incorporate individualized knowledge of parents into the plan of care. Engage HCPs in specific training regarding the care needs of CMC (e.g., analgesic requirements in children with developmental disability).
Communication practices	Prioritize timely delivery of clear and accurate information. Employ active listening skills to ensure parent concerns are heard and understood. Improve team communication to minimize conflicting messages and smooth transition between home/community and hospital.
Hospital setting and environment	Where possible, tailor hospital routines and policies to meet the individual needs of CMC/parents. Recognize the disruptive nature of the hospital environment on children and families. Support families in trying to ‘normalize’ their children's lives and routines during hospitalizations.

## 4 Discussion

The aim of this scoping review was to map the existing literature on the experiences of inpatient care for CMC and their families, describe the various aspects and methodologies and identify knowledge gaps. This review specifically pertains to the experience of care as a ‘user’ of healthcare services [20, 37]. CMC and their families who participated in these studies reported varied experience of care in relation to their ‘ideal’ state. Through qualitative and quantitative methods, participants described the importance of aspects such as communication, relationships, merging expertise of CMC, their families and HCPs and the significant impacts of the hospital setting.

These results confirm and reinforce the value that CMC and their families place critical elements during hospitalizations such as role negotiation, SDM and goal sharing. Relational elements of care are paramount, which is consistent with evidence from other healthcare settings and populations [28, 36]. In this review, relationships with HCPs, developing and incorporating expertise from both patient/parent and HCP and clear communication were highlighted as essential to enabling positive experiences for CMC and their families. Healthcare system factors not only influence these relational elements but also contribute to the environment that can be restrictive, generic and unsupportive.

Many of the concepts described in this scoping review are also central tenets to the practice of patient‐ and family‐centred care (PFCC). PFCC is the gold standard in delivering paediatric care and is intended to facilitate partnerships with patients and families [86]. PFCC emphasizes the importance of incorporating patient/family knowledge, good communication practices, collaborative decision‐making and ensuring that the physical environment is supportive [23]. PFCC is associated with improved quality of care by increasing satisfaction, reducing stress, improving patient knowledge and self‐management skills and even reducing hospital length of stay [87, 88]. Alarmingly, the themes identified in this review that corresponded closely to PFCC were often the practices that were most deficient from the perspective of CMC and their families. This suggests a knowledge‐to‐practice gap in the implementation of PFCC, at least to this population of CMC and their families. Providing PFCC to CMC in hospital is further complicated by a lack of evidence‐based models of care as demonstrated by our review which found only four interventional studies. These were further limited by small sample sizes, single‐institution design and lack of a control group. Future research should focus on developing and evaluating inpatient interventions for CMC that would target and address the experience of care outlined in this review alongside clinical, economic and patient‐oriented outcomes.

One of the objectives of this review was to identify gaps in the literature. There is a relative lack of quantitative studies even though validated instruments are available to measure experience of care concepts. Of the studies that collected quantitative data, the majority used non‐validated survey instruments that limit their rigour and prevent replication and comparison with other studies [89]. There is an overall lack of interventional studies, in particular studies that employ high levels of patient and family engagement (such as co‐design) and integrate patient‐ and family‐oriented outcomes into their evaluation framework. These types of studies may have the greatest potential to influence care experiences for CMC and their families.

The other potential gap is whether this evidence has captured all the essential aspects of experience of care for CMC/parents. Some authors approached their research with research questions pertaining to specific elements such as SDM [41, 54, 69], whereas others sought to describe the experience of care in a more general sense [56, 59, 61, 82]. The existing research likely captured many important issues for CMC and their families, but it remains possible that some crucial aspects were missed. Another consideration is whether the populations in these studies are representative of the population of CMC and their families at large. Demographic characteristics of study participants were reported but often did not include variables that would identify racialized minorities or gender diverse individuals. Those with limited English language proficiency were almost invariably excluded. Similarly, fathers were under‐represented in these studies. Thus, this description of experience of care may not reflect the perspectives of a diverse CMC population.

### 4.1 Limitations and Strengths

There are limitations inherent to this review. Methodological decisions made in the design and conduct of this review likely influenced our results in significant ways. For instance, we selected a definition of ‘experience of care’ that focused on ‘user’ or consumer‐related experiences, although other definitions would have included a broader range of experiences. This was done to ensure a feasible and meaningful synthesis and to best address our objectives.

A large majority of the research was conducted in the United States and only English language publications were included limiting extrapolation to other countries and populations. There may also be subpopulations of CMC based on demographics, diagnosis, neurodevelopmental disability or care needs, who have unique experience of care that are not captured fully in this review. Even with these limitations, this comprehensive review of hospital care for CMC synthesizes research from more than two decades, covering a wide variety of patient and family experiences. Inclusion of a parent–partner with lived experience of parenting a child with medical complexity (FB) ensured that our review objectives were relevant and that our results and discussion reflected the patient and family perspective. This review also identified pragmatic strategies that can be employed immediately by HCPs and institutions to address the experience of care for CMC and their families.

## 5 Conclusion

CMC and their families describe achieving an ‘ideal’ experience of care through negotiating care roles, decisions and goals, as well as relationship‐building, communication, merging the expertise brought by CMC, their families and professionals, and adapting the hospital environment to better meet their needs. Developing and testing interventions to improve the experience of care for CMC and their families are imperative to advancing this field of knowledge and improving the care that CMC receive in hospital.

## Author Contributions

**Tammie Dewan:** conceptualization, investigation, methodology, formal analysis, supervision, writing–original draft. **Lyndsay Mackay:** conceptualization, investigation, writing–review and editing, methodology, formal analysis. **Lauren Asaad:** investigation, writing–review and editing. **Francine Buchanan:** conceptualization, investigation, writing–review and editing, methodology, formal analysis. **K. Alix Hayden:** investigation, methodology, writing–review and editing, formal analysis, data curation. **Lara Montgomery:** investigation, writing–review and editing, formal analysis.

## Conflicts of Interest

The authors declare no conflicts of interest.

## Supporting information

Supporting information.

## Data Availability

The data that support the findings of this review are available from the corresponding author upon reasonable request.
