# Supplementary material for: Experiences of Inpatient Healthcare Services Among Children With Medical Complexity and Their Families: A Scoping Review
Source: Health Expect. 2024 Sep 4;27(5):e14178. doi: 10.1111/hex.14178 (PMC11372467; doi:10.1111/hex.14178)
Supplement: Supplementary file 1 — Supporting information. [file HEX-27-e14178-s001.docx]

**FINAL SEARCHES**

**Experiences of inpatient healthcare services among children with medical complexity:
A scoping review**

April 26, 2022

Database(s): **Ovid MEDLINE(R) and Epub Ahead of Print, In-Process, In-Data-Review & Other Non-Indexed Citations and Daily**1946 to April 25, 2022
Search Strategy:

| **#** | **Searches** | **Results** |
| --- | --- | --- |
| 1 | Medically at-risk.tw,kf. | 68 |
| 2 | (complex adj4 (chronic condition* or health condition* or medical condition* or care need* or medical need* or healthcare need* or health care need* or health need* or disabilit*)).tw,kf. | 3956 |
| 3 | (medical* adj2 (complex* or fragile*)).tw,kf. | 6089 |
| 4 | (health adj2 (complex* or fragile*)).tw,kf. | 5093 |
| 5 | (Technolog* adj2 dependen*).tw,kf. | 713 |
| 6 | (ventilator adj2 (dependent* or assist*)).tw,kf. | 1705 |
| 7 | (mechanical* adj2 assist* adj2 ventilat*).tw,kf. | 483 |
| 8 | mechanically ventilated.tw,kf. | 12772 |
| 9 | medically fragile technology dependent*.tw,kf. | 4 |
| 10 | (special health care need* or special healthcare need*).tw,kf. | 1857 |
| 11 | polyhandicap*.tw,kf. | 22 |
| 12 | (profound adj4 (intellectual or disabilit*)).tw,kf. | 1094 |
| 13 | (life-limiting adj2 (condition* or disabilit*)).tw,kf. | 649 |
| 14 | chronic critical illness.tw,kf. | 273 |
| 15 | (intellectual disabilit* adj5 hospital*).tw,kf. | 122 |
| 16 | or/1-15 | 32653 |
| 17 | Inpatients/ | 26925 |
| 18 | Hospitalization/ | 127174 |
| 19 | Child, Hospitalized/ | 7155 |
| 20 | exp Hospitals/ | 302686 |
| 21 | Adolescent, Hospitalized/ | 481 |
| 22 | Hospitals, Pediatric/ | 14761 |
| 23 | patient readmission/ | 21400 |
| 24 | Intensive Care Units, Pediatric/ | 9058 |
| 25 | ((paediatric or pediatric) adj2 (icu or icu* or intensive care or ward* or unit*)).tw,kf. | 19065 |
| 26 | (picu or picus).tw,kf. | 6252 |
| 27 | inpatient*.tw,kf. | 127173 |
| 28 | (hospital* adj2 patient*).tw,kf. | 116530 |
| 29 | hospital*.tw,kf. | 1494038 |
| 30 | acute care.tw,kf. | 25722 |
| 31 | or/17-30 | 1701859 |
| 32 | child/ or child, preschool/ or child/ or child, preschool/ | 2070859 |
| 33 | exp pediatrics/ | 62361 |
| 34 | (pediatric* or paediatric* or child* or preschool* or pre-school* or schoolchild* or schoolboy* or schoolgirl* or toddler* or boy or boys or girl* or pubescen* or juvenile* or teen* or youth* or high school* or adolesc* or pre-pubesc* or prepubesc*).tw,kf. | 2170463 |
| 35 | or/32-34 | 3000261 |
| 36 | 16 and 31 and 35 | 2714 |
| 37 | limit 36 to yr="2000 -Current" | 2443 |

Database(s): **Embase**1974 to 2022 April 25
Search Strategy:

| **#** | **Searches** | **Results** |
| --- | --- | --- |
| 1 | Medically at-risk.tw,kf. | 74 |
| 2 | (complex adj4 (chronic condition* or health condition* or medical condition* or care need* or medical need* or healthcare need* or health care need* or health need* or disabilit*)).tw,kf. | 5411 |
| 3 | (medical* adj2 (complex* or fragile*)).tw,kf. | 9887 |
| 4 | (health adj2 (complex* or fragile*)).tw,kf. | 6051 |
| 5 | (Technolog* adj2 dependen*).tw,kf. | 933 |
| 6 | (ventilator adj2 (dependent* or assist*)).tw,kf. | 2519 |
| 7 | (mechanical* adj2 assist* adj2 ventilat*).tw,kf. | 723 |
| 8 | mechanically ventilated.tw,kf. | 19708 |
| 9 | medically fragile technology dependent*.tw,kf. | 6 |
| 10 | (special health care need* or special healthcare need*).tw,kf. | 2198 |
| 11 | polyhandicap*.tw,kf. | 59 |
| 12 | (profound adj4 (intellectual or disabilit*)).tw,kf. | 1560 |
| 13 | (life-limiting adj2 (condition* or disabilit*)).tw,kf. | 978 |
| 14 | chronic critical illness.tw,kf. | 398 |
| 15 | (intellectual disabilit* adj5 hospital*).tw,kf. | 180 |
| 16 | or/1-15 | 47521 |
| 17 | hospital patient/ or hospitalized adolescent/ or hospitalized child/ | 209808 |
| 18 | hospital/ or pediatric hospital/ or hospitalization/ | 845345 |
| 19 | hospital readmission/ | 82655 |
| 20 | pediatric intensive care unit/ | 10454 |
| 21 | ((paediatric or pediatric) adj2 (icu or icu* or intensive care or ward* or unit*)).tw,kf. | 31826 |
| 22 | (picu or picus).tw,kf. | 13890 |
| 23 | inpatient*.tw,kf. | 214868 |
| 24 | (hospital* adj2 patient*).tw,kf. | 207978 |
| 25 | hospital*.tw,kf. | 2272249 |
| 26 | acute care.tw,kf. | 36443 |
| 27 | or/17-26 | 2662339 |
| 28 | child/ or juvenile/ or boy/ or girl/ or school child/ or adolescent/ | 2936052 |
| 29 | preschool child/ or toddler/ | 593795 |
| 30 | pediatrics/ | 85919 |
| 31 | (pediatric* or paediatric* or child* or preschool* or pre-school* or schoolchild* or schoolboy* or schoolgirl* or toddler* or boy or boys or girl* or pubescen* or juvenile* or teen* or youth* or high school* or adolesc* or pre-pubesc* or prepubesc*).tw,kf. | 2702862 |
| 32 | or/28-31 | 3938137 |
| 33 | 16 and 27 and 32 | 4935 |
| 34 | limit 33 to yr="2000 -Current" | 4633 |
| 35 | limit 34 to conference abstracts | 1716 |
| 36 | 34 not 35 | 2917 |

Database(s): **APA PsycInfo**1806 to April Week 3 2022
Search Strategy:

| **#** | **Searches** | **Results** |
| --- | --- | --- |
| 1 | chronically ill children/ | 415 |
| 2 | Medically at-risk.tw,id. | 53 |
| 3 | (complex adj4 (chronic condition* or health condition* or medical condition* or care need* or medical need* or healthcare need* or health care need* or health need* or disabilit*)).tw,id. | 1703 |
| 4 | (medical* adj2 (complex* or fragile*)).tw,id. | 1327 |
| 5 | (health adj2 (complex* or fragile*)).tw,id. | 1896 |
| 6 | (Technolog* adj2 dependen*).tw,id. | 280 |
| 7 | (ventilator adj2 (dependent* or assist*)).tw,id. | 80 |
| 8 | (mechanical* adj2 assist* adj2 ventilat*).tw,id. | 4 |
| 9 | mechanically ventilated.tw,id. | 242 |
| 10 | medically fragile technology dependent*.tw,id. | 2 |
| 11 | (special health care need* or special healthcare need*).tw,id. | 805 |
| 12 | polyhandicap*.tw,id. | 21 |
| 13 | (profound adj4 (intellectual or disabilit*)).tw,id. | 1285 |
| 14 | (life-limiting adj2 (condition* or disabilit*)).tw,id. | 235 |
| 15 | chronic critical illness.tw,id. | 19 |
| 16 | (intellectual disabilit* adj5 hospital*).tw,id. | 124 |
| 17 | or/1-16 | 7617 |
| 18 | hospitalized patients/ or patients/ | 40240 |
| 19 | hospitalization/ | 8113 |
| 20 | hospitals/ | 16348 |
| 21 | hospital admission/ | 3116 |
| 22 | intensive care/ | 4799 |
| 23 | ((paediatric or pediatric) adj2 (icu or icu* or intensive care or ward* or unit*)).tw,id. | 1227 |
| 24 | (picu or picus).tw,id. | 478 |
| 25 | inpatient*.tw,id. | 53874 |
| 26 | (hospital* adj2 patient*).tw,id. | 17412 |
| 27 | hospital*.tw,id. | 171819 |
| 28 | acute care.tw,id. | 5265 |
| 29 | or/18-28 | 233465 |
| 30 | pediatrics/ | 28672 |
| 31 | (pediatric* or paediatric* or child* or preschool* or pre-school* or schoolchild* or schoolboy* or schoolgirl* or toddler* or boy or boys or girl* or pubescen* or juvenile* or teen* or youth* or high school* or adolesc* or pre-pubesc* or prepubesc*).tw,id. | 1074888 |
| 32 | or/30-31 | 1075324 |
| 33 | 17 and 29 and 32 | 505 |
| 34 | limit 33 to yr="2000 -Current" | 450 |

**CINAHL Plus with Full Text (Ebsco)**

Search modes: Find all my search terms

| **#** | **Query** | **Results** |
| --- | --- | --- |
| S1 | (MH "Child, Medically Fragile") | 1,179 |
| S2 | TI “Medically at-risk” OR AB “Medically at-risk” | 111 |
| S3 | TI ( (complex N4 (“chronic condition*” or “health condition*” or “medical condition*” or “care need*” or “medical need*” or “healthcare need*” or “health care need*” or “health need*” or disabilit*)) ) OR AB ( (complex N4 (“chronic condition*” or “health condition*” or “medical condition*” or “care need*” or “medical need*” or “healthcare need*” or “health care need*” or “health need*” or disabilit*)) ) | 3,237 |
| S4 | TI ( (medical* N2 (complex* or fragile*)) ) OR AB ( (medical* N2 (complex* or fragile*)) ) | 3,513 |
| S5 | TI ( (health N2 (complex* or fragile*)) ) OR AB ( (health N2 (complex* or fragile*)) ) | 4,847 |
| S6 | TI (Technolog* N2 dependen*) OR AB (Technolog* N2 dependen*) | 536 |
| S7 | TI ( (ventilator N2 (dependent* or assist*)) ) OR AB ( (ventilator N2 (dependent* or assist*)) ) | 891 |
| S8 | TI (mechanical* N2 assist* N2 ventilat*) OR AB (mechanical* N2 assist* N2 ventilat*) | 169 |
| S9 | TI “mechanically ventilated” OR AB “mechanically ventilated” | 5,128 |
| S10 | TI “medically fragile technology dependent*” OR AB “medically fragile technology dependent*” | 4 |
| S11 | TI ( (“special health care need*” or “special healthcare need*”) ) OR AB ( (“special health care need*” or “special healthcare need*”) ) | 1,599 |
| S12 | TI polyhandicap* OR AB polyhandicap* | 5 |
| S13 | TI ( (profound N4 (intellectual or disabilit*)) ) OR AB ( (profound N4 (intellectual or disabilit*)) ) | 839 |
| S14 | TI ( (“life-limiting” N2 (condition* or disabilit*)) ) OR AB ( (“life-limiting” N2 (condition* or disabilit*)) ) | 549 |
| S15 | TI “chronic critical illness” OR AB “chronic critical illness” | 150 |
| S16 | TI (“intellectual disabilit*” N5 hospital*) OR AB (“intellectual disabilit*” N5 hospital*) | 121 |
| S17 | S1 OR S2 OR S3 OR S4 OR S5 OR S6 OR S7 OR S8 OR S9 OR S10 OR S11 OR S12 OR S13 OR S14 OR S15 OR S16 | 20,286 |
| S18 | (MH "Inpatients") OR (MH "Ventilator Patients") | 87,006 |
| S19 | (MH "Infant, Hospitalized") OR (MH "Child, Hospitalized") OR (MH "Adolescent, Hospitalized") | 6,226 |
| S20 | (MH "Hospitals") OR (MH "Hospitals, Pediatric") | 76,202 |
| S21 | (MH "Readmission") | 15,903 |
| S22 | (MH "Intensive Care Units, Pediatric") | 6,737 |
| S23 | TI ( ((paediatric or pediatric) N2 (icu or icu* or "intensive care” or ward* or unit*)) ) OR AB ( ((paediatric or pediatric) N2 (icu or icu* or "intensive care” or ward* or unit*)) ) | 9,860 |
| S24 | TI ( (picu or picus) ) OR AB ( (picu or picus) ) | 3,641 |
| S25 | TI inpatient* OR AB inpatient* | 60,182 |
| S26 | TI (hospital* N2 patient*) OR AB (hospital* N2 patient*) | 67,285 |
| S27 | TI hospital* OR AB hospital* | 529,105 |
| S28 | TI "acute care" OR AB "acute care" | 19,705 |
| S29 | S18 OR S19 OR S20 OR S21 OR S22 OR S23 OR S24 OR S25 OR S26 OR S27 OR S28 | 650,269 |
| S30 | (MH "Child") OR (MH "Child, Disabled") OR (MH "Child, Preschool") OR (MH "Minors (Legal)") | 572,578 |
| S31 | (MH "Adolescence") | 579,081 |
| S32 | (MH "Pediatrics") | 21,437 |
| S33 | TI ( (pediatric* or paediatric* or child* or preschool* or pre-school* or schoolchild* or schoolboy* or schoolgirl* or toddler* or boy or boys or girl* or pubescen* or juvenile* or teen* or youth* or “high school*” or adolesc* or pre-pubesc* or prepubesc*) ) OR AB ( (pediatric* or paediatric* or child* or preschool* or pre-school* or schoolchild* or schoolboy* or schoolgirl* or toddler* or boy or boys or girl* or pubescen* or juvenile* or teen* or youth* or “high school*” or adolesc* or pre-pubesc* or prepubesc*) ) | 802,584 |
| S34 | S30 OR S31 OR S32 OR S33 | 1,208,859 |
| S35 | S17 AND S29 AND S34 | 2,017 |
| S36 | S17 AND S29 AND S34  Limiters - Published Date: 20000101-20221231 | 1,862 |

SRMedicalFragile Web of Science Search

Web of Science Core Collection

Editions = A&HCI , ESCI , CPCI-SSH , CPCI-S , SCI-EXPANDED , SSCI

#1

**“Medically at-risk” or “mechanically ventilated” or “medically fragile technology dependent*” or “special health care need*” or “special healthcare need*” or polyhandicap* or “chronic critical illness”** (Topic) or **(complex Near/4 (“chronic condition*” or “health condition*” or “medical condition*” or” care need*” or “medical need*” or “healthcare need*” or “health care need*” or “health need*” or disabilit*))** (Topic) or **(medical* Near/2 (complex* or fragile*))** (Topic) or **(health Near/2 (complex* or fragile*))** (Topic) or **(Technolog* Near/2 dependen*)** (Topic) or **(ventilator Near/2 (dependent* or assist*))** (Topic) or **(mechanical* Near/2 assist* Near/2 ventilat*)** (Topic) or **(profound Near/4 (intellectual or disabilit*))** (Topic) or **(life-limiting Near/2 (condition* or disabilit*))** (Topic) or **(“intellectual disabilit*” Near/5 hospital*)** (Topic)

#2

**((paediatric or pediatric) Near/2 (icu or icu* or “intensive care” or ward* or unit*))** (Topic) or **(picu or picus or inpatient*)** (Topic) or **(hospital* Near/2 patient*)** (Topic) or **hospital* or "acute care"** (Topic)

#3

**(pediatric* or paediatric* or child* or preschool* or pre-school* or schoolchild* or schoolboy* or schoolgirl* or toddler* or boy or boys or girl* or pubescen* or juvenile* or teen* or youth* or “high school*” or adolesc* or pre-pubesc* or prepubesc*)** (Topic)

**#2 AND #3 AND #1**

#2 AND #3 AND #1 and 2021 or 2022 or 2020 or 2019 or 2018 or 2017 or 2016 or 2015 or 2014 or 2013 or 2011 or 2012 or 2000 or 2001 or 2002 or 2003 or 2004 or 2005 or 2006 or 2007 or 2008 or 2009 or 2010 (Publication Years)
